# Suspected Miscarriage in the Experience of Emergency Medical Services Teams—Preliminary Study

**DOI:** 10.3390/ijerph182312305

**Published:** 2021-11-23

**Authors:** Ewa Rzońca, Agnieszka Bień, Grażyna Bączek, Patryk Rzońca, Michał Filip, Robert Gałązkowski

**Affiliations:** 1 Department of Obstetrics and Gynecology Didactics, Faculty of Health Sciences, Medical University of Warsaw, 14/16 Litewska St., 00-575 Warsaw, Poland; gbaczek@wum.edu.pl; 2 Chair of Obstetrics Development, Faculty of Health Sciences, Medical University of Lublin, 4-6 Staszica St., 20-081 Lublin, Poland; agnieszka.bien@umlub.pl; 3 Department of Human Anatomy, Faculty of Health Sciences, Medical University of Warsaw, 5 Chałubińskiego St., 02-004 Warsaw, Poland; przonca@wum.edu.pl; 4 Department of Obstetrics and Pathology of Pregnancy, Medical University of Lublin, 16 Staszica St., 20-081 Lublin, Poland; michalfilip@umlub.pl; 5 Department of Emergency Medical Services, Faculty of Health Sciences, Medical University of Warsaw, 14/16 Litewska St., 00-575 Warsaw, Poland; robert.galazkowski@wum.edu.pl

**Keywords:** Emergency Medical Service, vaginal bleeding, miscarriage, women

## Abstract

Vaginal bleeding and abdominal pain are symptoms indicative of a threat to pregnancy that prompt women to seek assistance from health care professionals. The purpose of the study was to present the characteristics of Emergency Medical Services (EMS) team interventions in cases of suspected miscarriage. The study involved a retrospective analysis of EMS team interventions in cases of suspected miscarriage carried out between January 2018 and December 2019 in Poland. Data obtained from Poland’s National Monitoring Center of Emergency Medical Services included emergency medical procedure records and EMS team dispatch records in electronic format. The mean patient age was 30.53 years. Most were primiparous (48.90%) and up to the 13th gestational week (76.65%). The most commonly reported symptom was vaginal bleeding (80.71%). EMS teams were most commonly dispatched in the winter (27.03%), between 7 A.M. and 6:59 P.M. (51.87%), in urban areas (69.23%), with urgency code 2 (55.60%), and in most cases, they transferred the patient to a hospital (97.53%). The present study addresses very important issues concerning the characteristics of Polish suspected miscarriage cases handled by different EMS team types, in different locations (urban vs. rural areas), and concerning patients in a different obstetric situation (gestational week, gravidity, parity). Our findings suggest a need for further studies in this field and for gestational health promotion activities to be implemented, specifically including actions to reduce the risk of vaginal bleeding during pregnancy.

## 1. Introduction

Symptoms such as spotting or bleeding, amniotic fluid leakage, abdominal pain, or lower back pain may be indicative of a miscarriage [1,2], or early spontaneous pregnancy loss, whereby a live or dead embryo or fetus is passed before 22 weeks of pregnancy [3]. Vaginal bleeding is one alarming symptom occurring in many early pregnancies, which may precede a miscarriage [4,5]. In addition, the literature features many reports of the impact of a woman’s psychological health on her experience of pregnancy loss. Notably, there is no single reaction to a miscarriage common to all women. Research demonstrates that women who had lost a pregnancy may experience sadness, shame, or guilt, in addition to symptoms of depression, grief, or anxiety, which may be transient or persist for a number of years. All of the above factors may affect the woman’s health or the course of subsequent pregnancies [6,7,8,9]. 

As vaginal bleeding and abdominal pain are symptoms indicative of a threat to pregnancy, they prompt women to seek assistance from health care professionals, including via hospital emergency departments [1,7,9,10]. Importantly, these and other alarming symptoms may occur at any time during pregnancy and constitute a threat both to the woman’s health and life [11,12]. In light of the constitutional duty to provide medical assistance to anyone who needs it, the National Emergency Medical Services system was founded in Poland. Its main task is to provide help to people experiencing a medical emergency posing an immediate risk to their life or health. The system is based on two independent pillars, i.e., Hospital Emergency Wards and Emergency Medical Services (EMS), including Polish Medical Air Rescue crews. To fulfill the State’s constitutional duty to provide medical assistance to everyone who needs it, the Polish Emergency Medical Services (EMS) system was established. The main task of the EMS is to provide care to patients with severe or life-threatening emergencies. The system comprises hospital emergency departments and EMS teams, including air medical rescue teams. EMS teams are units within the system that are responsible for responding to emergencies and performing the necessary medical emergency procedures directly on scene and during patient transport to hospital. EMS teams are composed of physicians, emergency medical technicians, and nurses, and Polish EMS teams are classified as specialist, or physician-staffed, and basic, or non-physician-staffed [13].

The purpose of the study was to present the characteristics and to analyze interventions of EMS teams in cases of suspected miscarriage. 

## 2. Materials and Methods

The present study comprised a retrospective analysis of Emergency Medical Services team interventions regarding suspected miscarriage in Polish women, conducted on the basis of data from the National Monitoring Center of Emergency Medical Services for the period of January 2018–December 2019. This is a national ICT system allowing for accepting emergency calls and event notifications from emergency numbers, dispatching EMS teams, logging medical events, including their location, preparing medical documentation, and managing calls [14]. Data obtained from the system included electronic records, namely emergency medical procedure documentation and EMS team dispatch documentation. The following information was obtained from the electronic records: date of call, location of call, details regarding the pregnant patient, clinical parameters of the patient, ICD–10 diagnosis codes assigned (International Statistical Classification of Diseases and Related Health Problems), emergency medical procedures applied, and other information on the interventions. The study received approval from the Polish Ministry of Health, which also supplied data for analysis. The Bioethics Committee of the Medical University of Warsaw in Poland reviewed the study protocol and confirmed that the retrospective nature of the study did not necessitate consent (statement AKBE/104/2021).

Cases classified under the following ICD–10 codes by the EMS team were analyzed: O03—spontaneous abortion, O03—other abortion, O06—unspecified abortion; where the gestational age did not exceed 22 weeks. We used the following exclusion criteria: medical assistance refusal, call cancellation, patient’s absence on site, and gestational age above 22 weeks. Information on the gestational week was generated from the EMS medical documentation data which, in turn, are based on the information that EMS team members obtain from pregnant women and/or from the maternity notes. Of 2152 EMS team interventions in cases of suspected miscarriage, 1820 cases were ultimately included in the analysis based on the established criteria. 

A statistical analysis of the data was conducted using the STATISTICA software, v. 13.2 (Tibco Software Inc., Palo Alto, CA, USA). Numbers (n) and percentages (%) were used to describe qualitative data, and means (M) and standard deviations (SD) were used to report quantitative data. The Kolmogorov–Smirnov test and the Lilliefors test were used to analyze the normality of distribution for quantitative variables. The chi^2^ statistic was used to test statistically significant differences between qualitative variables. The non-parametric Mann–Whitney U-test was used to test differences between two independent groups and the Kruskal–Wallis test was used for more than two groups. Furthermore, the Spearman correlation coefficient was calculated. The significance level adopted in the study was *p* < 0.05.

## 3. Results

The mean age of the women studied was 30.53 years. Most patients were primiparous (48.90%), up to the 13th gestational week (76.65%), and had no history of miscarriage (91.32%). The most reported symptom was vaginal bleeding (80.71%). Detailed data are shown in Table 1.

Table 2 shows the characteristics of EMS team interventions in cases of suspected miscarriage. The interventions occurred most often in the winter (27.03%), between 7 A.M. and 6:59 P.M. (51.87%), in urban areas (69.23%). Most interventions were assigned urgency code 2 (55.60%) and were carried out by a three-person (52.20%), non-physician-staffed EMS team (59.12%). The emergency medical procedure most commonly provided by EMS team members to the pregnant patients was blood pressure measurement (94.84%), and further management typically involved patient transfer to hospital (97.53%).

Our analysis of associations between EMS team type and selected variables showed that non-physician-staffed EMS teams were more often dispatched with urgency code 2, with three responders per team. In addition, in non-physician-staffed team interventions, the pregnant patient remained on scene for a longer time, and the total duration was longer than in the case of interventions by physician-staffed EMS teams. An analysis of EMS calls to pregnant patients with suspected miscarriage from rural areas demonstrated that the patients tended to be older, and more interventions were assigned urgency code 1. Patients from rural areas were also more often transported to hospital by the EMS team, and intervention duration was longer than in urban areas. These findings were statistically significant (*p* < 0.05)—see Table 3.

The statistical analysis conducted revealed a statistically significant association between the gestational week in the women with suspected miscarriage, to whom EMS teams were dispatched, and the history of miscarriage and urgency code (*p* < 0.05). Furthermore, there was a statistically significant association between the number of pregnancies and the number of deliveries in the women studied and their age, history of miscarriage, location of call, urgency code, and EMS team type (*p* < 0.05)—Table 4.

The analysis showed a positive correlation between the gestational week and the number of pregnancies, the number of deliveries, and heart rate and respiration rate values. In contrast, there was a weak correlation between the gestational week and the saturation value. The analysis also showed a strong positive correlation between the number of pregnancies and the number of deliveries, and a weak positive correlation between the number of pregnancies and the MAP value (mean arterial pressure). Furthermore, there was a weak positive correlation between the number of deliveries and the MAP value. The above-mentioned correlations were statistically significant (*p* < 0.05). Detailed data are presented in Table 5.

## 4. Discussion

Pregnancy loss and its impact, mainly on the psychological health of women, represents a major challenge for the public health sector and health care professionals providing care to patients in this exceptional time [7,15]. In addition, patients experiencing symptoms that can potentially indicate a threat to the pregnancy may seek assistance by various means: reporting to a clinic or a hospital emergency department, or placing an emergency call [1,4,9,10,13]. These aspects motivated us to investigate the characteristics of EMS team interventions in cases of suspected miscarriage, or pregnancy loss up to the 22nd gestational week. 

In Ontario, most pregnant patients seen at the emergency department were under 25 years old, nulliparous, and in the first trimester of pregnancy [16]. In a study by Miller et al. (2019), women reporting at the emergency department with a miscarriage were mostly under 30, pregnant for a second or subsequent time, had given birth at least once before, and had a history of miscarriage [8]. In our study, EMS teams were dispatched mainly to women with suspected miscarriage who were 30 years old on average, primiparous, in the first trimester, and had no history of miscarriage. The most commonly reported symptom was vaginal bleeding. Notably, vaginal bleeding in early pregnancy occurs in approximately one in four pregnant women, and half of these cases end in miscarriage [4,5]. Baird et al. (2018) reported the experiences of women with miscarriage in an emergency room. The most common symptom reported by the patients was spotting, and most patients sought assistance due to concerns about the associated health risk [17]. In turn, a Turkish study demonstrated that in women who experienced bleeding in pregnancy, the bleeding was most commonly described as minor. Most patients initially waited for it to stop, and only later sought help at an emergency department [18]. 

Cox et al. (2020) presented a study on the early pregnancy care model. In the analyzed period, between July 2018 and February 2019, 25 pregnant patients were transported from Early Pregnancy Assessment Units to the John Radcliffe hospital, mainly due to ectopic pregnancy or heavy bleeding. The number of transports was the highest in July [19]. According to our findings, EMS team interventions concerning cases of suspected pregnancy loss most often took place in the winter, during the daytime, with urgency code 2, and in urban areas. A study by Wierzbik-Strońska et al. (2020) on the characteristics of EMS team interventions in southern Poland also demonstrated that the teams were most often dispatched to patients with various health problems during the prenatal, perinatal, and postpartum periods in urban areas [20]. In turn, Strehlow et al. (2016) and Bills et al. (2018) reported that most EMS interventions for pregnant patients were performed in rural areas [21,22]. Similarly, Varner et al. (2020) reported that most pregnant patients seen at the emergency department were rural residents [16]. Furthermore, Strehlow et al. (2016) found that the emergency medical procedures performed by EMS team members most commonly included the measurement of primary vital signs—heart rate, blood pressure, and respiration rate [21], which is similar to our findings, with blood pressure measurement being performed in most cases. It needs to be noted that the study material indicates that medical emergency procedures, such as the measurement of primary vital signs, were not carried out in all the pregnant women. This may suggest that, indeed, these procedures were not performed or that this aspect was not included in the medical documentation filled in by EMS team members due to the need for urgent transport of the patient to hospital. Nevertheless, this situation shows that EMS team members should complete medical records with greater diligence. The results of our study revealed that the more advanced the gestational week, the higher the number of previous deliveries and pregnancies, and the higher the heart rate and respiration rate values. Conversely, the more advanced the pregnancy, the lower the saturation value. The analysis also demonstrated that a higher number of pregnancies was associated with a higher number of deliveries and a higher MAP value, and that a higher number of previous deliveries was associated with a higher MAP value obtained in the examination performed by EMS. In our study, the mean duration of EMS team interventions for pregnant patients was also longer when the teams were dispatched to rural areas than to urban ones, which is similarly corroborated by Strehlow et al. (2016) [21]. Analyzing the health problems of pregnant women requiring EMS assistance, Freitas et al. (2020) found that the most common reason for the call was labor, followed by miscarriage, preeclampsia, and vaginal bleeding. They also reported that multiparous women and those in the third trimester of pregnancy were more likely to call Mobile Emergency Care Services [23]. By comparison, our study demonstrated that in the case of rural areas, EMS teams were more often dispatched to women in the third or subsequent pregnancy and those who had a history of three or more deliveries.

One important aspect of pregnancy loss involves its risk factors. Among the many risk factors for miscarriage, researchers have highlighted the mother’s age, socio-economic standing, nutritional status, and substance use; history of obstetric problems such as miscarriage, premature labor, cesarean section, or gestational diabetes mellitus; performing physical labor requiring heavy lifting; stress; infections; and hormonal factors [24,25,26,27]. Both maternal age above 35 years and multiple previous deliveries increase the risk of miscarriage [24]. We found that the number of EMS team interventions was related to the age of the woman, gestational age, the number of pregnancies, and the number of deliveries. 

Our study is the first retrospective analysis of all calls registered by the National Monitoring Center of Emergency Medical Services in Poland, which has allowed for gathering reliable data on EMS team interventions in cases of women with suspected miscarriage. Moreover, it complements our previous study on vaginal bleeding in pregnant women as a reason for EMS team intervention [28]. 

Our analyses showed that EMS teams were more often dispatched to primiparous women, in the third trimester of pregnancy, nulliparous women, and women who experienced miscarriage, and that the dominating symptoms were vaginal bleeding and abdominal pain. Interestingly, pregnant women living in rural areas were assigned code 1, which means that an urgent dispatch of an EMS team was required, with the shortest possible time of reaching the location of the event, because a health-threatening emergency requires an immediate use of medical emergency procedures. A threat to pregnancy requires immediate action. If the patient is at home, this means calling the EMS, followed by rapid transport to hospital and further professional management. It is estimated that about 10–15% of all pregnancies in Poland end with miscarriage. Data collected based on reports about the general activity of hospitals indicate that in Poland the number of women who miscarried in 2017 was over 40,000, in 2018—nearly 39,000, and in 2019—over 39,000. Looking at the scale of the miscarriage prevalence, it is crucial to apply immediate interventions focused on maintaining the pregnancy [29]. 

This study has certain limitations. The analysis only includes information from the EMS electronic medical records. We do not have information on the follow-up care provided to the patients after they had been transferred to hospital or on patient condition in cases where the woman was left in place. Furthermore, we do not have any knowledge on the patient health or obstetric status with regard to the suspected miscarriage after the EMS team interventions have taken place, which makes it impossible to analyze the entire process of care provided to these women. However, these limitations do not affect the quality of the study performed.

The results of our study indicate that it is necessary to conduct further research on health problems in pregnant women with suspected miscarriage to better understand the issue in question. Furthermore, research should be conducted in the area of EMS team members’ preparation to provide assistance to pregnant women, especially in cases of high-risk pregnancies. As a result, pregnant women will receive better care, including that provided by EMS team members who are responsible for offering medical assistance on site.

## 5. Conclusions

EMS teams most commonly intervened in cases of pregnant women with suspected miscarriage who were at a mean age of 30 years, primiparous, and in the first trimester of pregnancy. The most reported symptom was vaginal bleeding. Most interventions were carried out in the winter, during the daytime, in urban areas, with urgency code 2, and included patient transport to hospital. 

The present study addresses very important issues concerning the characteristics of Polish suspected miscarriage cases handled by different EMS team types (physician-staffed vs. non-physician staffed), in different areas (urban vs. rural area), and concerning patients in a different obstetric situation (gestational week, gravidity, parity). It points to a need for further studies in this field and for gestational health promotion activities to be implemented, specifically including actions to reduce the risk of early pregnancy loss.

## Figures and Tables

**Table 1:** Characteristics of the women with suspected miscarriage.

Age—M (SD)	30.53 (6.76)
Number of pregnancies—n (%)	
1st	890 (48.90)
2nd	393 (21.59)
3rd or more	537 (29.51)
Number of pregnancies—M (SD)	2.08 (1.42)
Gestational week—n (%)	
≤13 weeks	1395 (76.65)
≥14 weeks	425 (23.35)
Gestational week—M (SD)	10.79 (4.15)
Number of previous deliveries—n (%)	
0	916 (50.33)
1–2	438 (24.07)
3 or more	466 (25.60)
Number of previous deliveries—M (SD)	1.43 (1.70)
History of miscarriage—n (%)	
Yes	158 (8.68)
No	1662 (91.32)
Symptoms—n (%)	
Bleeding	1469 (80.71)
Abdominal pain	792 (43.52)
Spotting	220 (12.09)
Contractions	161 (8.85)
Vomiting	60 (3.30)
Diarrhea	19 (1.04)

**Table 2:** Characteristics of EMS team interventions.

Time of Year—n (%)	
Spring	438 (24.07)
Summer	452 (24.84)
Fall	438 (24.07)
Winter	492 (27.03)
Time of call—n (%)	
7 A.M.–6.59 P.M.	944 (51.87)
7 P.M.–6.59 A.M.	876 (48.13)
Location of call—n (%)	
Urban area	1260 (69.23)
Rural area	560 (30.77)
Urgency code—n (%)	
Code 1	808 (44.40)
Code 2	1012 (55.60)
EMS team type—n (%)	
Non-physician-staffed	1076 (59.12)
Physician-staffed	744 (40.88)
EMS team composition—n (%)	
Two-person	870 (47.80)
Three-person	950 (52.20)
Medical emergency procedures—n (%)	
Blood pressure measurement	1726 (94.84)
Pulse oximetry	1686 (92.64)
Physical examination	1267 (69.62)
Intravenous cannulation	939 (51.59)
Blood glucose measurement	511 (28.08)
Intravenous medication	439 (24.12)
Fluid therapy	428 (23.52)
Analgesic treatment	97 (5.33)
Gynecological examination	81 (4.45)
Selected physical examination findings—M (SD)	
Heart rate	94.12 (17.06)
Mean arterial pressure	99.68 (14.76)
Respiration rate	16.05 (4.18)
Saturation	97.93 (1.21)
Blood glucose	114.64 (37.10)
^1^ GCS	14.96 (0.57)
^2^ RTS	11.95 (0.30)
Further management—n (%)	
Patient transferred to hospital	1775 (97.53)
Patient left in place	45 (2.47)
Total intervention duration (min)—M (SD)	40.82 (16.59)

^1^ GCS—Glasgow Coma Scale; ^2^ RTS—Revised Trauma Score.

**Table 3:** Analysis of the relationship between EMS team type and call location, and selected variables.

Variables	EMS Team Type		*p*-Value	Location of Call		*p*-Value
	Non-Physician-Staffed	Physician-Staffed		Urban Area	Rural Area	
Age—M (SD)	30.57 (6.94)	30.47 (6.51)	0.9229	30.29 (6.85)	31.06 (6.53)	0.0283
Time of call—n (%)						
7 A.M.–6.59 P.M.	564 (52.42)	380 (51.08)	0.5735	663 (52.62)	281 (50.18)	0.3362
7 P.M.–6.59 A.M.	512 (47.58)	364 (48.92)		597 (47.38)	279 (49.82)	
Location of call n (%)						
Urban area	757 (70.35)	503 (67.61)	0.2122	-	-	-
Rural area	319 (29.65)	241 (32.39)		-	-	
Urgency code—n (%)						
Code 1	417 (38.75)	391 (52.55)	0.0000	537 (42.62)	271 (48.39)	0.0221
Code 2	659 (61.25)	353 (47.45)		723 (57.38)	289 (51.61)	
EMS team composition—n (%)						
Two-person	820 (76.21)	50 (6.72)	0.0000	597 (47.38)	273 (48.75)	0.5894
Three-person	256 (23.79)	694 (93.28)		663 (52.62)	287 (51.25)	
Further management—n (%)						
Assistance provided, patient transferred to hospital	1041 (96.75)	734 (98.66)	0.0099	1222 (96.98)	553 (98.75)	0.0251
Patient left in place	35 (3.25)	10 (1.34)		38 (3.02)	7 (1.25)	
Intervention duration (min)—M (SD)	42.55 (16.88)	38.35 (15.87)	0.0000	36.77 (15.40)	49.80 (15.59)	0.0000

**Table 4:** Analysis of the relationship between gestational week, number of pregnancies and number of previous deliveries and selected variables.

Variables	Gestational Week		*p*-Value	Number of Pregnancies			*p*-Value	Number of Previous Deliveries			*p*-Value
	≤13 Weeks	14 or More		1st	2nd	3rd or More		None	1–2	3 or More	
Age—M (SD)	30.57 (6.86)	30.39 (6.44)	0.6413	29.38 (7.13)	30.03 (6.24)	32.78 (5.92)	0.0000	29.47 (7.10)	30.06 (6.25)	33.04 (5.86)	0.0000
History of miscarriage—n (%)											
Yes	94 (6.74)	64 (15.06)	0.0000	0 (0.00)	40 (10.18)	118 (21.97)	0.0000	40 (4.37)	71 (16.21)	47 (10.09)	0.0000
No	1301 (93.26)	361 (84.94)		890 (100.00)	353 (89.82)	419 (78.03)		876 (95.63)	367 (83.79)	419 (89.91)	
Location of call—n (%)											
Urban area	975 (69.89)	285 (67.06)	0.2678	651 (73.15)	278 (70.74)	331 (61.64)	0.0000	673 (73.47)	301 (68.72)	286 (61.37)	0.0000
Rural area	420 (30.11)	140 (32.94)		239 (26.85)	115 (29.26)	206 (38.36)		243 (26.53)	137 (31.28)	180 (38.63)	
Urgency code—n (%)											
Code 1	565 (40.50)	243 (57.18)	0.0000	357 (40.11)	189 (48.09)	262 (48.79)	0.0015	374 (40.83)	206 (47.03)	228 (48.93)	0.0073
Code 2	830 (59.50)	182 (42.82)		533 (59.89)	204 (51.91)	275 (51.21)		542 (59.17)	232 (52.97)	238 (51.07)	
EMS team type—n (%)											
Non-physician-staffed	841 (60.29)	235 (55.29)	0.0668	555 (62.36)	222 (56.49)	299 (55.68)	0.0221	574 (62.66)	247 (56.39)	255 (54.72)	0.0073
Physician-staffed	554 (39.71)	190 (44.71)		335 (37.64)	171 (43.51)	238 (44.32)		342 (37.34)	191 (43.61)	211 (45.28)	

**Table 5:** Analysis of the correlation between gestational week, number of pregnancies and number of previous deliveries and selected variables.

Variables	Gestational Week		*p*-Value	Number of Pregnancies			*p*-Value	Number of Previous Deliveries			*p*-Value
	≤13 Weeks	14 or More		1st	2nd	3rd or More		None	1–2	3 or more	
Number of pregnancies	r = 0.182		0.0000	-			-	-			-
Number of previous deliveries	r = 0.159		0.0000	r = 0.945			0.0000	-			-
Selected physical examination findings											
Heart rate	r = 0.068		0.0048	r = 0.020			0.3963	r = 0.010			0.6910
MAP	r = 0.040		0.0978	r = 0.072			0.0031	r = 0.057			0.0196
Respiration rate	r = 0.055		0.0225	r = 0.026			0.2857	r = 0.024			0.3257
Saturation	r = −0.058		0.0185	r = 0.004			0.8836	r = 0.004			0.8819

## Data Availability

The data presented in this study are available on request from the corresponding author.
